# Modified-Malay Eating Behavior and Pattern Questionnaire (Malay-EBPQ): translation and validation among Malaysian women

**DOI:** 10.1186/s12955-023-02182-2

**Published:** 2023-08-29

**Authors:** Premaa Supramaniam, Siti Hajar Ismail, Aisyah Ali, E- Li Leong, Prema Muninathan, Tassha Hilda Adnan, Sarala Paramesvaran

**Affiliations:** 1 Clinical Research Center (CRC), Level 4, Ambulatory Care Centre (ACC) Building, Raja Permaisuri Bainun Hospital, Jalan Raja Ashman Shah, Ministry of Health, Ipoh, Perak 30450 Malaysia; 2 Dietetic Department, Hospital Umum Sarawak, Ministry of Health, Kuching, Sarawak Malaysia; 3 Clinical Research Centre (CRC), Hospital Sultan Ismail, Ministry of Health, Johor Bahru, Johor Malaysia; 4 Clinical Research Centre (CRC), Hospital Taiping, Ministry of Health, Taiping, Perak Malaysia; 5 Department of Statistics Malaysia, Putrajaya, Malaysia; 6 Sekolah Jenis Kebangsaan (Tamil), Ministry of Education, Perai, Penang Malaysia

**Keywords:** Validity, Reliability, Eating behavior, Pattern, Translation, Women

## Abstract

**Background:**

Eating behavior primarily depends on eating patterns which are largely influenced by interactions between physiology, environment, psychology, culture and socio-economic status. This study was designed to translate and validate the Eating Behavior Pattern Questionnaire (EBPQ) among Malaysian women.

**Methods:**

A cross-sectional study involving translation and validation of the English version of EBPQ. The original questionnaire, contained 51 items extracted into six domains was translated in Malay using forward and backward translation, pre-tested and validated among conveniently sampled female healthcare personnel. Vegetarians, pregnant ladies and women in confinement were excluded due to special daily dietary plans. Construct validity, reliability and feasibility were analyzed using Exploratory Factor Analysis (EFA) and Confirmatory Factor Analysis (CFA).

**Results:**

During translation, item modifications were made and subjected to field testing among 394 women. The original questionnaire was used as a reference to identify the positioning of items in constructs. Fifteen items were removed due to poor correlation with items within constructs. Seven factors were extracted using Varimax rotation with Kaiser–Meyer–Olkin (KMO) value range from 0.725–0.872 and significant Bartlett’s test of Sphericity (*p* < 0.001). The item-loading of the items within the constructs ranged between 0.415–0.812 (explained variation = 62.7%). C*ultural and lifestyle behavior* was relabeled to *lifestyle and behavioral eating*, and *snacking on sweets* was relabeled as *snacking pattern*. *Emotional eating* was divided into two sub-factors as *snacking behavior* and *emotional influence*. CFA resulted with an acceptable fit with no presence of floor and ceiling effects. Intra-class correlation coefficient (ICC) for all the constructs were reported good and excellent. The overall internal consistency was reported as good.

**Conclusion:**

The modified 36-item Malay-EBPQ had moderate internal consistency, reliable and fit with multi-dimensional measures of eating behaviors and dietary patterns among women in the multi-racial population with cultural diversity.

## Introduction

Eating behavior whether healthy or unhealthy eating is based on what food to eat, when to eat and how much to eat. Eating behavior primarily depends on eating patterns which largely influenced by interactions between physiology, environment, psychology, culture, socio-economic status and genetics [1]. In the past decades, much research has focused on these independent influential factors on the eating behavior but multi-disciplinary researches are required to explain the complexity of the interaction of these influential factors [2, 3].

On the other hand, nutritional epidemiology field classically focused on the nutrients. Dietary patterns, essential elements for promoting good health, revolve around the composition and quantity of nutrients found in different food types [2, 3]. The most recent Dietary Guidelines underscore the significance of shifting dietary pattern analysis from a singular focus on individual nutrients to a more comprehensive understanding of the intricate interplay between nutrients and food consumption. This approach takes into account not only the types of food and nutrients but also their quantities and consumption frequencies [2, 3].

Furthermore, dietary intake varies among individuals. Notably, dietary patterns exhibit a more pronounced association with psychological well-being among women compared to men. Women are particularly sensitive to changes in dietary patterns, which can disrupt their healthy eating habits [4]. Triggered by various factors, alterations in eating patterns indirectly stimulate and influence mental well-being and mood. This relationship gains significance when considering the impact of exercise patterns. Additionally, the quality of protein exerts a noteworthy influence on women's dietary patterns, surpassing its impact on men's dietary choices [4].

Thus, it is essential for a specific tool to evaluate the eating patterns taking into account of the eating behavioral and dietary patterns while targeting different genders. Eating Behavior Pattern Questionnaire (EBPQ) was designed based on African-American women population to assess the eating behavior and patterns with 51 items and six domains. EBPQ was mainly developed to incorporate measures of emotional eating and snacking habit apart of dietary fat and fiber intake. It measures the eating pattern relevant to health outcomes and diseases prevention but not based on dietary assessment on specific food and portion sizes estimation [5].

Dehghan et al. translated and validated EBPQ among Iranian female Tabriz University student. The domains of the Persian-EBPQ was expended into nine constructs with additional domains of healthy eating and eating out. Considering the cross-cultural translation, two items were changed based on Iranian cultural adjustment where the terms of *vending machine* was changed to *fast-food restaurant* and *church socials* was changed to charities [6].

EBPQ has segmented the multi-dimensional measures of eating behaviors among female population which makes it suitable for testing eating behavior and patterns in a multi-racial population with cultural diversity [5, 7, 8]. Thus, this study was conducted to translated EBPQ in Malay and validated it among female adult population in Malaysia. The Malay-EBPQ will be useful for nutritional assessment particularly on dietary behavioral assessment of fat and fiber intake in general female Malaysian population and Malay language was established as the National language in the multi-ethnic country [9].

## Materials and methods

### Subjects and study design

**Fig. 1:**
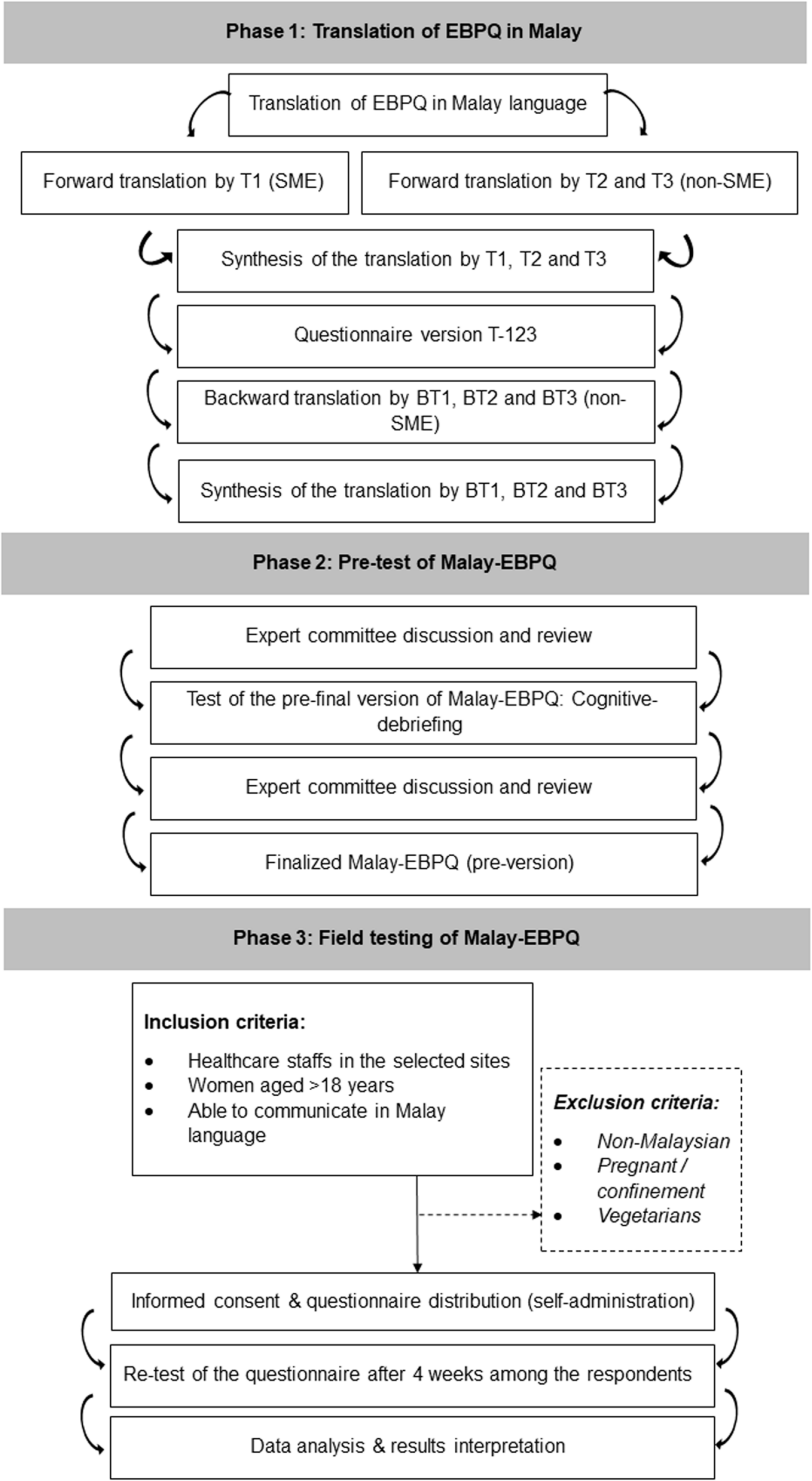
Translation & validation process of Malay-EBPQ

This was a cross-sectional study involving conveniently sampled female healthcare personnel under the Ministry of Health (MOH) in Peninsular Malaysia. The study included four tertiary referral hospitals and the National Institutes of Health (NIH), with data collection taking place in December 2020. Female adults who are able to communicate in Malay were considered eligible for the study. Vegetarian (ovo-lacto vegetarian, ovo-vegetarian, lacto-vegetarian and vegan) [10], pregnant ladies and woman in confinement were excluded due to special daily dietary plans. Recruitments were targeted at the admission area, non-critical wards and administrative offices. This study was conducted in three phases in accordance with the guidelines for the process of cross-cultural adaptation of self-report measures and as illustrated in Fig. 1 [11].

### Phase 1 (Translation of Malay-EBPQ)

Phase 1 was the translation process of English version of EBPQ into Malay language by using internationally accepted translation process which was adopted from the International Society for Quality of Life Assessment (IQOLA) project [11–13]. The translation process was conducted by two independent groups of individuals for forward and backward translation.

T1 is the subject-matter expert from medical background and was aware of the concept of the questionnaire. T1 contributed more of clinical perspective during forward translation. Whereas, T2 and T3 were the non-subject matter experts from the non-medical background and unaware of the concepts of the questionnaire. T2 and T3 emphasized the laymen language used in the population. All T1, T2 and T3 were independently involved in forward translation and had synthesized the translated version of EBPQ via a discussion and thus finalized the translated version as EBPQ version T-123.

Then, BT1, BT2 and BT3 were the three independent non-medical background personnel involved in the backward translation of EBPQ version T-123. They were blinded on the original version of EBPQ.

Post-translation, there was a discussion among the expert committee to finalize the Malay-EBPQ. The expert committee consisted of the translators (T1, T2, T3, BT1, BT2 and BT3), study investigators, health professional representatives (dietitian, nutritionist, medical officer and pharmacist) and language professionals.

### Phase 2 (Pre-testing for content validity)

Phase 2 of the study consisted of two pre-tests of the questionnaire. The first pre-test was conducted among 10 conveniently sampled female adults after passing the bilingual competency test. Individual interviews were conducted to obtain the respondents’ feedback on the comprehension of the Malay-EBPQ through cognitive debriefing. The expert committee reviewed the comments and suggested appropriate amendments.

The second pre-testing of the questionnaire was conducted among a sample size of 30 additional randomly selected female adults in two chosen study sites. This number of participants, which is widely accepted for such studies, was deemed sufficient to ensure adequate evaluation and validation of the questionnaire's content and structure [14]. This group of respondents were given the Malay-EBPQ for further testing. Post-analysis, the final version of Malay-EBPQ questionnaire was finalized by the expert committee.

### Phase 3 (Field testing for construct validity and reliability)

To test the validity and reliability, the questionnaire was tested among targeted population by trained data collectors. Eligible candidates were identified and consented participants had administered the Malay-EBPQ via e-questionnaire using a QR code in 30 min.

Participants repeated the procedure of answering the Malay-EBPQ four weeks apart for test–retest analysis as per recommended 1–4 weeks as sufficient interval for test–retest reliability testing [15]. Respondents were required to use anonymous alpha-numeric code during the first and second questionnaire administration for matching purposes. Demographic details of age, race and educational level were also collected.

Bujang et al. suggested for a minimum of five to seven respondents for each item in a questionnaire is required if the construct validity is fairly moderate with no other issues. Thus, a minimum of 396 samples were required with an additional 10% dropout rate [16].

### Study instrument

The original English version of the EBPQ was developed by Schlundt et al. [5]. It was designed as a culturally specific eating behavior pattern questionnaire to predict the intake of total fat and fiber among African-American women. This tool was developed based on the Kristal Eating Patterns Questionnaire (KEPQ) and a 16-item Eating Styles Questionnaire (ESQ) and reported with good internal consistency [5].

The questionnaire contained 51 items with six constructs; *low-fat eating* (14 items), *emotional eating* (10 items), *snacking on sweets* (6 items), *cultural / lifestyle behaviors* (7 items), *haphazard planning* (9 items) and *meal skipping* (5 items). The items were scored based on 5-point Likert Scale measurement from *strongly agree* to *strongly disagree*. This tool was validated among females with average age of 40 years and with average Body Mass Index (BMI) of 29.8 kg/m^2^. EBPQ was reported with good internal consistency, reliability and construct validity with internal consistency coefficient ranges from 0.50 to 0.84 [5].

### Statistical analysis

Statistical analysis was performed using IBM SPSS (Version 28.0) and AMOS (AMOS Development Corporation). *P*-value of less than 0.05 was considered as statistically significant.

### Demographic characteristics of the respondents

Mean and standard deviation (SD) were used to describe the demographic characteristics of the respondents for continuous data, whereas frequency and percentages (%) were used for categorical data.

### Construct validity

Exploratory Factor Analysis (EFA) was performed to identify the underlying structure (construct validity) of the Malay-EBPQ as compared to the original English version of EBPQ. Eigenvalue greater than 0.10 was used. Correlation between the items was investigated using Barlett’s test of Sphericity while Kaiser–Meyer–Olkin (KMO) was used to measure the sample adequacy. Factor loading of 0.4 or more was considered good [17].

Confirmatory Factor Analysis (CFA) was performed to test the fit of the constructs of Malay-EBPQ. Fit Indices of *X*^*2*^/df ratio less than three, Comparative Fit Index (CFI) greater than 0.95, and Root Mean Square Error (RMSEA) less than 0.06 were used to justify the fit of the model [18, 19].

### Reliability

For reliability analysis, internal consistency and test–retest reliability were used. Cronbach’s alpha of more than 0.5 was acceptable and a value of more than 0.7 was considered as good [20]. The corrected item-to-total correlation of more than 0.3 was acceptable [21]. Intra-class correlation coefficient (ICC) with value of 0.40 and less was considered as poor to fair, 0.41–0.60 as moderate, 0.61–0.80 as good and greater than 0.80 as excellent [22, 23].

### Feasibility

A floor effect occurs when several respondents score the lowest possible score, whereas a ceiling effect occurs when several respondents score the highest possible score. The amount of ceiling and floor effects determine the quality of the content validity. Ceiling and floor effects were evaluated to be satisfactory by analyzing the percentages of the scores at the extremes of the response scale to be less than 15% [24, 25].

## Results

### Demographic characteristics

A total of 394 (99.2%) completed questionnaires were included in the analysis. Two responses were excluded as the respondents withdrew participation. Respondents were female MOH healthcare personnel with tertiary (78.9%) and secondary (20.3%) education with mean 37.8 (SD: 7.84) years of age. Malay (84.5%) respondents were majority (Table 1).

**Table 1:** Socio-demographic characteristics of the respondents (*n* = 394)

Socio-demographic characteristics	Frequency (%)
Gender—Female	394 (100.0)
Age, (years) [mean (SD)]	37.8 (7.84)
Ethnicity	
Malay	333 (84.5)
Chinese	35 (8.9)
Indian	22 (5.6)
Others (*Bajau, Banjar, Dusun & Iban)*	4 (1.0)
Highest educational level	
Tertiary	311 (78.9)
Secondary	80 (20.3)
Primary	3 (0.8)

### Content validity

The Malay-EBPQ was translated into Malay by two groups of subject-matter experts and non-subject-matter experts using forward and backward translations. The translated version was reviewed and finalized by the expert committee.

Several items were changed where words and phrases were modified during translation process. Phrases like *beef and pork* and *church socials* were changed to *beef, mutton and pork* and *religious gathering*.

Additional substitute questions were created with the intention of maintaining the true meaning of the original statement but using alternative words that carry the same meaning. The statement *When I buy snack foods, I eat until I have finished the whole package* was added with an additional substitute statement by replacing the statement with a passive structure. Similarly, the statement *When I buy snack foods, I eat until I have finished the whole package* was added with a substitute with a much shorter statement but preserved the meaning of the original statement. Both the original and substitutes question was subjected for final testing. The substitute questions were selected due to better construct validity.

The original constructs as in the original questionnaire were used as a reference to identify the positioning of the translated items in the respective construct. Items were removed from the construct when the item-to-total correlation is less than 0.3 as illustrated in Table 2. Thirteen items were removed due to poor correlation within the construct. However, additional two more items *I eat meatless meals from time to time because I think that is healthier for me* and *I try to limit my intake of red meat (beef and pork)* have been removed from the *Low-fat Eating* construct as these items were unable to integrate with other items in the same construct even though the correlation values are 0.311 and 0.394 (Table 2). The remaining 36 items were subjected to construct validity and reliability testing.

**Table 2:** Eliminated items in Malay-EBPQ

Questionnaire items (15 items)	Original construct	Item-total- correlation
I eat at religious gatherings.	Cultural/lifestyle behaviors	0.149
I take time to plan meals for the coming week.		0.156
When I am upset, I tend to stop eating.	Emotional eating	-0.266
I eat for comfort.		0.262
My eating habits are very routine.	Haphazard planning	-0.019
I have at least three to four servings of vegetables per day.		0.004
I stop for a fast food breakfast on the way to work.		0.109
I would rather buy takeout food and bring it home and cook.		0.146
I never know what I am going to eat for supper when I get up in the morning.		0.268
Instead of planning meals, I choose what is available and what I feel like eating.	Low-fat eating	-0.098
I buy snacks from vending machines.		-0.077
I like to eat vegetables seasoned with fatty meat.		-0.009
Fish and poultry are the only meats I eat.		0.225
I eat meatless meals from time to time because I think that is healthier for me.		0.311
I try to limit my intake of red meat (beef, mutton and pork).		0.394

### Construct validity

EFA of the 36 items resulted with seven factor extraction using Varimax rotation with KMO value range from 0.725 to 0.872 and significant Bartlett’s test of Sphericity (*p* < 0.001). Varimax rotation was used since the constructs are uncorrelated and the rotation minimizes the number of items that have higher factor loadings on each construct [26, 27].

Two of the extracted contracts were considered as the sub-factors of a major contract of *emotional eating.* The item-loading of the items within the constructs ranged between 0.415 to 0.812 and the combination of the items within the constructs led to a maximum of 62.7% explained variation (Table 4-Appendix).

The extracted items in the constructs led to modification of the labelling of certain constructs. The original construct, *cultural and lifestyle behavior* has been modified to *lifestyle and behavioral eating*. Meanwhile, *snacking on sweet* has been changed to *snacking pattern*. *Emotional eating* has been divided into two sub-factors as *snacking behavior* and *emotional influence*.

**Fig. 2:**
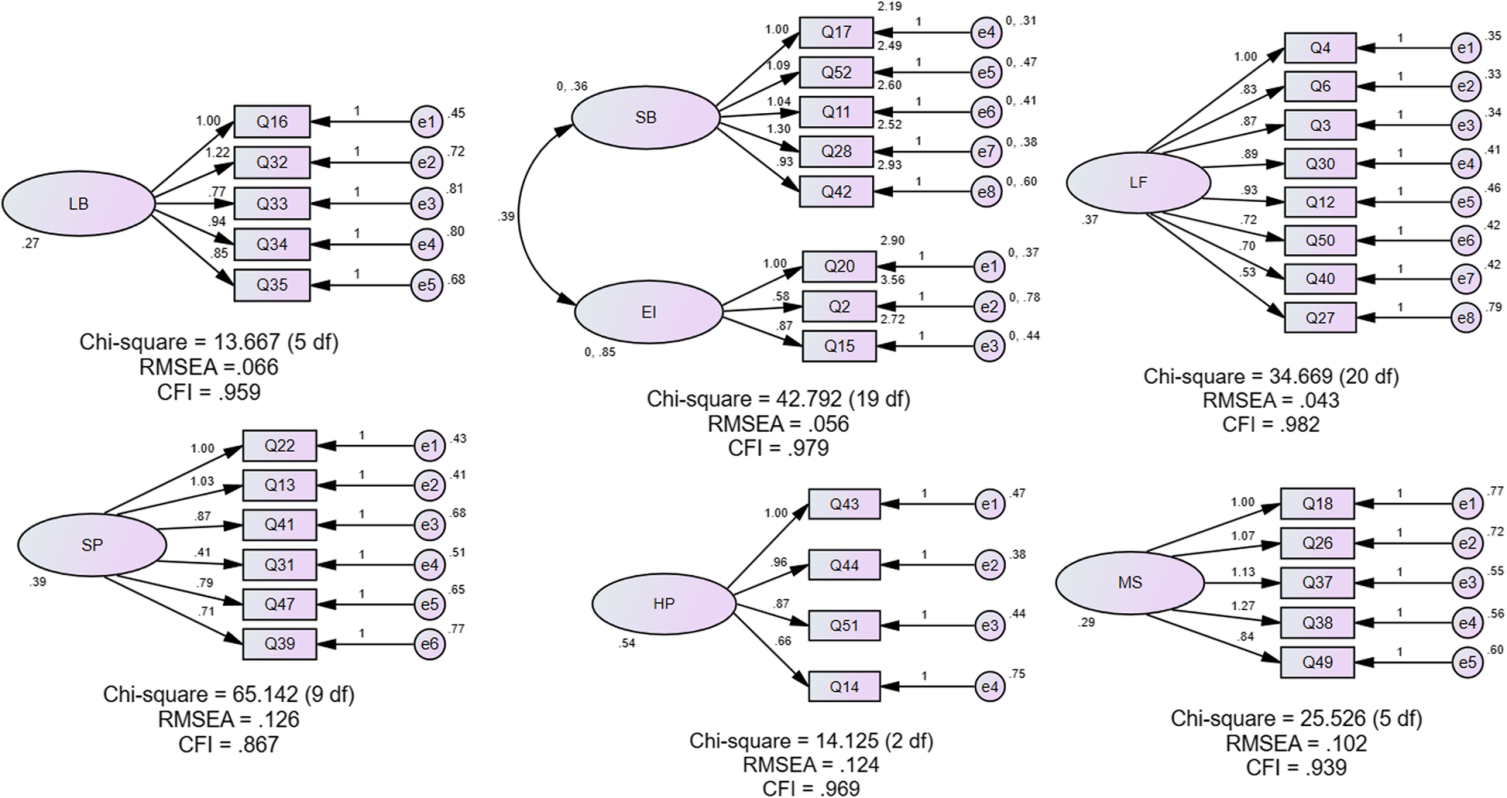
Structural Equation Modelling for constructs in the Malay-EBPQ (LB-Lifestyles and Behavioral eating, SP-Snacking patterns, SB-Snacking behavior, EI-Emotional influence, HP-Haphazard planning, LF-Low-fat eating, MS-Meal skipping)

CFA analysis of the seven constructs resulted in an acceptable fit of the modified version of the questionnaire as shown in Fig. 2. Three constructs (*lifestyles and behavioral eating, emotional eating* and *low-fat eating*) were reported with good fit where the fit indices *X*^*2*^/df ratio, CFI and RMSEA reported within the cut-off values. On the other hand, the other constructs (*haphazard planning, meal skipping* and *snacking pattern*) were reported with unfit fit indices.

### Feasibility and reliability

There is no presence of floor and ceiling effects in all the constructs as it ranges from 0.3% to 2.0%. Whereas, ICC for all the constructs were reported good and excellent (range: 0.88–0.94) (Table 3).

**Table 3:** Feasibility and reliability of Malay-EBPQ

Constructs	Floor effect, n (%)	Ceiling effect, n (%)	ICC (95% CI)	Cronbach’s alpha (α)
LB: Lifestyle & behavioral eating	4 (1.0)	1 (0.3)	0.88 (0.76, 0.94)	0.639
EE: Emotional eating				
SB: Snacking behavior	8 (2.0)	1 (0.3)	0.88 (0.77, 0.94)	0.824
EI: Emotional influence	7 (1.8)	5 (1.3)	0.88 (0.77, 0.94)	0.745
HP: Haphazard planning	12 (3.0)	1 (0.3)	0.93 (0.86, 0.97)	0.759
LF: Low-fat eating	1 (0.3)	1 (1.3)	0.89 (0.78, 0.94)	0.812
MS: Meal skipping	4 (1.0)	1 (0.3)	0.94 (0.88, 0.97)	0.718
SP: Snacking pattern	1 (0.3)	1 (0.3)	0.92 (0.84, 0.96)	0.719
**Overall (36 items)**	1 (0.3)	1 (0.3)	0.93 (0.86, 0.96)	0.895

## Discussion

The study aimed to translate and validate the original EBPQ questionnaire in Malay language by considering the cross-cultural aspects of the multi-racial country. Taking into account the diversity of dietary patterns that are greatly influenced by ethnic diversity in Malaysia, several items were modified during the translation process.

*Beef* and *pork* reflect specific preferences in certain ethnic groups. Pork is favored among the Chinese but is non-halal in Islamic dietary guidelines [28]. Muslims prefer beef, while Hindus avoid it [29, 30]. Thus, the choice of words has been adjusted for understanding and acceptance in the multiracial society.

The original phrase *I eat at the church socials* does not capture diverse eating habits during *cultural* gatherings. Instead, *religious gathering* was used to encompass a holistic understanding of such gatherings in a pluralistic society. Similarly, the Persian version of EBPQ replaced *church socials* with *charities* to better fit the Iranian population [6].

Item selection was modified based on the original EBPQ [5]. Eleven items with low correlation and loading values (< 0.3) were removed. *Haphazard planning* lost five items, and *low-fat eating* lost four due to poor correlation within their constructs.

In the present study, questions related to *I eat meatless meals from time to time because I think that is healthier for me* and *I try to limit my intake of red meat (beef and pork)* were removed from the *low-fat eating* construct. These items failed to integrate with others, resulting in instability [31]. Misunderstanding about fat, influenced by meat types and preparation, contributed to the disconnect [32, 33]. Fat intake involves various dietary aspects, including cooking methods, oils and food choices [34].

Items in the *emotional eating* construct split into two sub-constructs. The items *When I am in a bad mood, I eat whatever I feel like eating* and *My emotions affect what and how much I eat* represented mood-related eating and negative emotions. These items resemble some questions in the Mood Eating Scale [35–38]. Impulsive women were more likely to engage in emotional eating during negative moods than women with low impulsivity [39].

Five inversely-worded questions with negative factor loadings were reported in the original 51-item EBPQ [5]. All of these questions were eliminated due to poor factor loading. Respondents took more time to explain their understanding during cognitive-debriefing. Briefly explained questions were less confusing for respondents [40]. Question number, length and positioning significantly influence response quality [41].

The final modified version of Malay-EBPQ had 36 items extracted into seven constructs with adequate samples and the items were strongly correlated with one another within constructs (KMO > 0.70, significant Bartlett’s test of Sphericity) [31, 42–44]. The internal consistency for all the constructs were considered good (Cronbach’s alpha: 0.72–0.82) except for *lifestyles and behavioral eating* (Cronbach’s alpha = 0.64). However, the overall internal consistency was good (Cronbach’s alpha = 0.90) with acceptable model fit together with solid constructs of ≥ 5 items with factor loading ≥ 0.05 [31, 45]. In contrary, previous studies have reported moderate consistency for *cultural/lifestyle* (Crobach’s alpha: 0.54–0.60) [5, 6]. Persian version had moderate consistency for *emotional eating* but reported with acceptable overall fit of nine constructs [6].

The translation of the EBPQ into Malay brings forth compelling strengths and implications. It enhances accessibility, cultural sensitivity and validity among the Malay-speaking population, enabling seamless cross-cultural comparisons. The availability of a Malay version empowers targeted public health interventions and personalized dietary advice, catered to the unique needs of the Malay-speaking community. Moreover, it fosters robust community engagement, highlighting the significance of diverse cultural perspectives in research. The translated questionnaire enhances scientific research by developing a deeper comprehension of eating habits in a multicultural setting.

However, this study was limited by the use of convenient sampling of women serving in healthcare facilities, which resulted in a skewed distribution of educational backgrounds, potentially restricting the representativeness of the women population in Malaysia. Moreover, the present study did not include assessments for convergent and discriminant validity. Therefore, in future studies, it is important to consider examining the external and discriminant validity of the modified version of the translated Malay-EBPQ. Discriminant validity could be targeted to compare the scores of different constructs between healthy women and women with extreme health outcomes. Additionally, larger-scale trials, involving diverse populations, comparison with established measures, and thorough analysis of psychometric properties, will be essential to ensure the tool's robustness and its ability to yield accurate and consistent results.

### Applications of the modified Malay-EBPQ

The developer of the EBPQ has highlighted the use of the questionnaire for individual evaluation, intervention assessment and research purposes that investigates the relationship between health outcomes and eating behavior [5]. The modified Malay-EBPQ can be used in the Malaysian population as Malay language was established as the National language in the multi-ethnic country.

## Conclusion

The translation of the English version of EBPQ demonstrated reduction of items based on cross-cultural adaptivity and item fitting within constructs. The modified 36-item Malay-EBPQ had six constructs with 2 sub-constructs, reported with moderate internal consistency, reliable and fit. Malay-EBPQ is useful as it composes of multi-dimensional measures of eating behaviors and dietary patterns assessment among women in the multi-racial population with cultural diversity.

## Data Availability

Data and materials used in the study for this manuscript are available from the corresponding author upon request. The translated version of questionnaire and the dataset generated for the current study are available from the corresponding author on reasonable request.

## Appendix

**Table 4:** Items factor loading within constructs of Malay-EBPQ

Items	Factor loading	Mean (SD)	Explained variation (%)
**Construct 1: Lifestyle & behavioral eating [KMO = 0.725]**		14.4 (3.12)	41.3
I buy meat every time I go to the grocery store.	0.701		
I have a serving of meat at every meal.	0.694		
On Sunday, I eat dessert more than once a day.	0.629		
A complete meal includes a meat, a starch, a vegetable, and bread.	0.627		
I associate success with food.	0.551		
**Construct 2: Emotional eating [KMO = 0.872]**		21.9 (5.44)	
**Construct 2a: Snacking behavior**		12.7 (3.54)	36.1
I snack more at night.	0.794		
When I buy snack foods, I eat until I have finished the whole package.	0.789		
I am a snacker.	0.764		
If I am bored, I will snack more.	0.684		
I sometimes snack even when I am not hungry.	0.612		
**Construct 2b: Emotional influence (3 items)**		9.2 (2.59)	62.7
When I am in a bad mood, I eat whatever I feel like eating.	0.783		
My emotions affect what and how much I eat.	0.773		
I eat when I am upset.	0.765		
**Construct 3: Haphazard planning [KMO = 0.738]**		10.1 (2.95)	58.5
I eat out because it is more convenient than eating at home.	0.812		
I hate to cook.	0.804		
I eat at a fast food restaurant at least three times a week.	0.789		
When I don't plan meals, I eat fast food.	0.643		
**Construct 4: Low-fat eating [KMO = 0.885]**		28.5 (4.38)	44.6
I carefully watch the portion sizes of my foods.	0.755		
I use low-fat food products.	0.716		
I count fat grams.	0.702		
I am very conscious of how much fat is in the food I eat.	0.704		
I choose healthy foods to prevent heart diseases.	0.717		
When choosing fast food, I pick a place that offers healthy foods.	0.641		
I reduce fat in recipes by substituting ingredients and cutting portions.	0.630		
I take a shopping list to the store.	0.415		
**Construct 5: Meal skipping [KMO = 0.755]**		14.3 (3.39)	47.1
If I am busy, I will eat a snack instead of lunch.	0.737		
If I eat a larger than usual lunch, I will replace supper with a snack.	0.709		
If I eat a larger than usual lunch, I will skip supper.	0.687		
I rarely eat breakfast.	0.649		
If I do not feel hungry, I will skip a meal even if it is time to eat.	0.644		
**Construct 6** **: ** **Snacking pattern [KMO = 0.742]**		17.4 (3.56)	42.0
I eat cookies, candy bars, or ice-cream in place of dinner.	0.739		
I snack two or three time every day.	0.720		
To me, cookies are an ideal snack food.	0.667		
I have a sweet tooth.	0.645		
Sometimes I eat dessert more than once a day.	0.587		
I usually keep cookies in the house.	0.498		

*Varimax rotated of Principle Axis Factoring; Bartlett’s test of Sphericity (p* < *0.001), Total mean (SD)* = *106.6 (15.60)*
